# Fecal biomarkers: Non-invasive diagnosis of colorectal cancer

**DOI:** 10.3389/fonc.2022.971930

**Published:** 2022-09-02

**Authors:** Qian Ding, Xiangxu Kong, Weilong Zhong, Wentian Liu

**Affiliations:** Department of Gastroenterology and Hepatology, Tianjin Medical University General Hospital, Tianjin Institute of Digestive Disease, Tianjin, China

**Keywords:** fecal biomarkers, diagnosis, CRC, non-invasive, screening

## Abstract

Colorectal cancer (CRC) is the third most common cancer in the world in terms of morbidity and mortality, which brings great health hazards and economic burdens to patients and society. A fecal examination is an effective method for clinical examination and the most commonly used method for the census. It is simple, non-invasive, and suitable for large-scale population screening. With the development of molecular biology, lots of efforts have been made to discover new fecal biomarkers for the early screening of colorectal cancer. In this review, we summarize and discuss the recent advances of fecal biomarkers for CRC screening or diagnosis, including DNA biomarkers, RNA biomarkers, protein biomarkers, gut microbes and volatile organic compounds focusing on their diagnostic evaluation for CRC, which can provide a basis for the further development of new and effective CRC fecal screening and early diagnosis techniques.

## 1 Introduction

The latest GLOBOCAN data showed that there were about 1.9 million new cases of colorectal cancer worldwide in 2020 ranking third in the global incidence of malignant tumors and there were about 935000 deaths, ranking second in the global malignant tumor deaths (1). The 2022 cancer report in China shows that the incidence of colorectal cancer in China has increased significantly and ranks second among all tumors. However, the 5-year survival rate of targeted early screening can reach more than 90% (2). In the United States, through efficient screening methods, the incidence and mortality of colorectal cancer have declined, and the 5-year survival rate has increased significantly (3).

**Figure 1:**
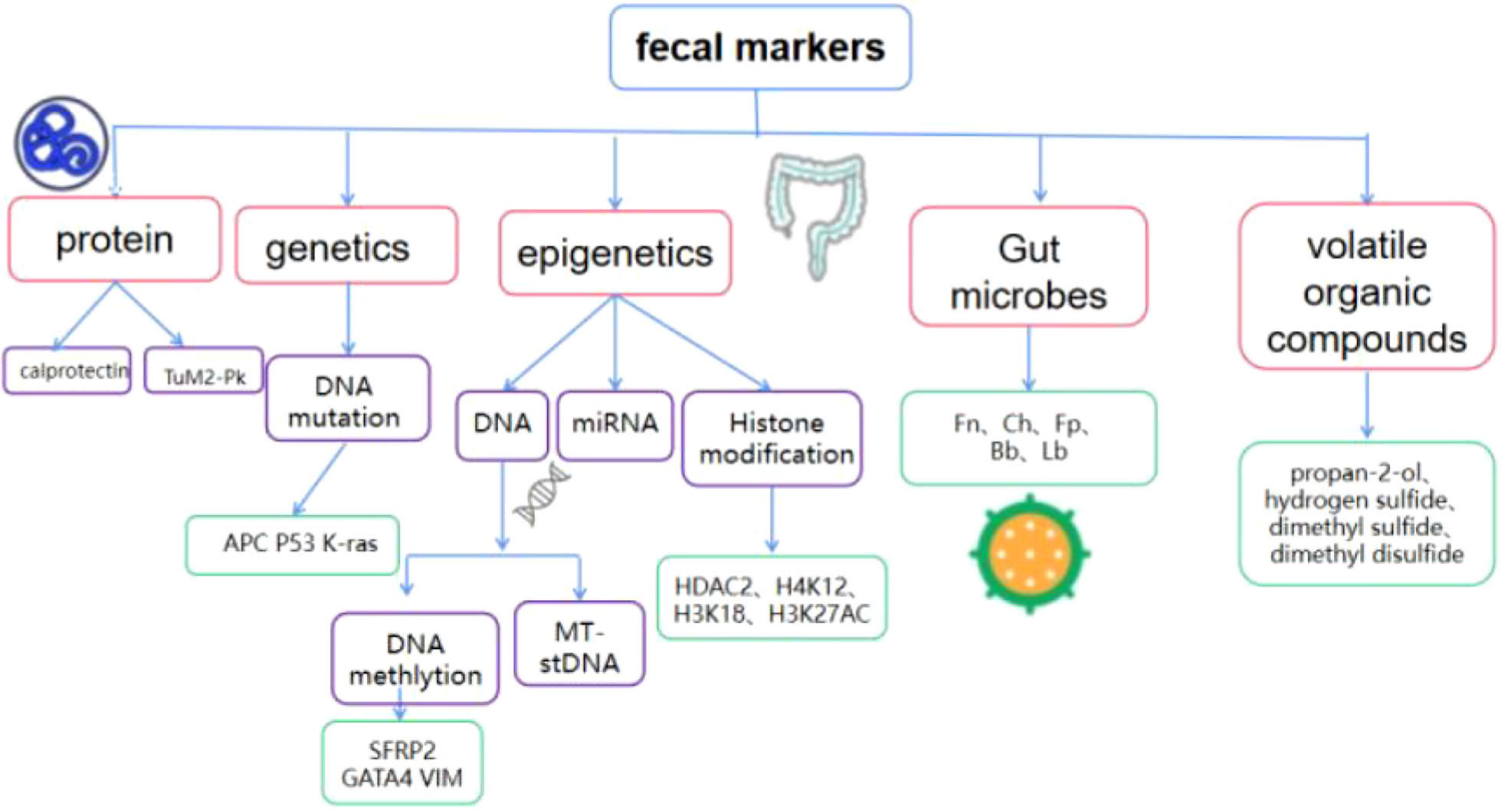
Composition of fecal biomarkers for colorectal cancer TuM2-Pk, Tumor M2 pyruvate kinase; SFRP2, secreted frizzled-related protein 2;APC, adenomatous polyposis; GATA4, GATA Binding Protein 4; VIM, vimentin; MT-sDNA, Multi-target stoolDNA; HDAC2, histone deacetylase 2; Fn, Fusobacterium nucleatum;Ch, Clostridium hathewayi;Fp, Faecalibacterium Prausnitzii;Bb, Bifidobacterium; Lb, Lactobacillus.

Currently, widely used screening methods include a digital rectal examination, stool examination, imaging examination, endoscopy, and tumor marker detection. Among them, imaging examinations are expensive and expose patients to medical radiation; endoscopy is an invasive examination, and patient compliance is poor. The fecal occult blood test (FOBT) is a widely accepted non-invasive test compared to colonoscopy. However, non-hemorrhagic colorectal tumors or polyps, and those that do not consistently drain enough blood into the lumen, cannot be detected by either guaiac fecal occult blood test (gFOBT) or immunochemical faecal occult blood test (iFOBT/FIT). The non-invasion, convenience, effectiveness, economy, and safety of fecal biomarkers can improve the overall acceptance of screening, and are an important method for colorectal cancer screening and diagnosis (4). The potential of DNA, RNA, proteins, microbes and volatile organic compounds in stool as biomarkers for colorectal cancer screening and early diagnosis has attracted much attention (**Figure 1**). These biomarkers have the characteristics of simple sampling and low risk and are expected to become a new target for the next generation of colorectal cancer screening and early diagnostic testing. This review will summarize the latest research progress on fecal biomarkers for colorectal cancer, and provide a theoretical reference for the development and evaluation of new technologies for subsequent colorectal cancer screening and early diagnosis.

## 2 Novel biomarkers

### 2.1 Protein biomarkers

#### 2.1.1 Calprotectin

Calprotectin is a calcium-binding and zinc-binding protein complex that is abundant in the cytoplasm of inflammatory cells. Fecal calprotectin (FC) concentration has a good correlation with intestinal inflammation. FC can be used as a marker of gastrointestinal diseases and has been used in the diagnosis of inflammatory bowel disease for many years. It aids in the diagnosis of inflammatory bowel disease and irritable bowel syndrome (5, 6). In recent years, with the deepening of the research on FC, the application of FC in CRC has gradually increased.

The results from Shi Haiyun et al. (7) showed that the FC level of patients with colorectal cancer was significantly higher than that of non-colorectal cancer patients, and the FC level increased with the severity of colorectal lesions. The sensitivity (73%) and negative predictive value (87%) of FC in diagnosing colorectal cancer were both high. The high sensitivity suggests that FC can also be a noninvasive marker for colorectal cancer screening. A high negative predictive value suggests that most patients with negative FC can rule out colorectal cancer. Tibble et al. (8) found that 90% of patients with colorectal cancer had elevated FC levels, but only 58% of patients with colorectal cancer had positive occult blood. The overall sensitivity and specificity of FC on colorectal cancer and adenomatous polyps as a combination group were 79% and 72%, respectively, while the sensitivity and specificity of fecal occult blood were 43% and 92%, respectively. It is suggested that the sensitivity of FC to detect colorectal cancer is higher than that of fecal occult blood. Relevant studies have shown that FC combined with other stool tests has more diagnostic value. One study showed that FC combined with FIT had a sensitivity of 80% and a specificity of 93% in the diagnosis of CRC (9). At present, because FC detection still has many shortcomings, such as low specificity, high detection cost, and complicated detection steps and operations, it is not listed as a routine CRC detection method. Given the special significance of FC detection for CRC and the deepening of related research, it is worth looking forward to FC becoming a diagnostic marker for CRC in the future.

#### 2.1.2 TuM2-PK

Tumor M2 pyruvate kinase (TuM2-PK) is a key enzyme in glycolysis and plays an important role in tumor pathogenesis. TuM2-PK is expressed at elevated levels in patients with many tumors, such as esophageal, gastric, pancreatic, and colorectal cancers (10, 11). In 2004 and 2006, researchers conducted pilot studies on TuM2-PK, and the results showed that TuM2-PK could be used as a biomarker for colorectal cancer screening (12–14). These encouraging results provided the basis for subsequent studies. One study performed iFOBT and quantitative ELISA stool testing for TuM2-PK in 127 patients. The study found that the combination of iFOBT and TuM2-PK can improve the diagnostic accuracy of pre-neoplastic and neoplastic colon lesions. Therefore, high levels of TuM2-PK enhance the efficacy of CRC screening tests (15). Another study suggested that fecal TuM2-PK positive has high sensitivity and diagnostic accuracy for colorectal cancer and colorectal precancerous lesions. Furthermore, in this study, the combined use of M2PK, iFOBT, and FC had a sensitivity and specificity of 95% and 47.5%, respectively, in detecting adenomas ≥ 1 cm in size. Therefore, the combined use of M2PK, iFOBT and FC may be valuable in the detection of macro adenomas (16). A quantitative analysis of 24 tumor biomarkers in colonic mucus indicated that TuM2-PK was one of the 4 tumor biomarkers with the highest sensitivity, specificity, and diagnostic efficacy for colorectal cancer (17). In addition, a systematic review and meta-analysis showed that for diagnosing colorectal cancer, the sensitivity and specificity of the stool test for M2-PK were 79% and 80%, respectively, with higher sensitivity than FOBT but lower specificity (18). All these studies demonstrate the important value and broad prospects of TuM2-PK in the early screening of colorectal cancer. For future studies, Hisham et al. (19) suggested that more clinical studies with larger samples are needed and passed and other marker comparisons were performed to better assess the value of TuM2-PK in screening for CRC, and patients were longitudinally followed up with TuM2-PK prognosis after treatment to assess survival.

### 2.2 Genetic and epigenetic related biomarkers

#### 2.2.1 Fecal DNA methylation and mutation

Growing evidence suggests that epigenetic changes are major tumor origin determinants and heterogeneity. Gene mutations and epigenetic modifications drive progression from normal mucosa to cancer by altering signaling pathways that regulate cancer behavior (20). A number of DNA methylation biomarkers have been found to be associated with CRC and precancerous lesions in stool samples (21). Fecal DNA tests for screening CRC have evolved significantly over time, and due to CRC-related molecular abnormalities and technological advances, we can better detect molecular abnormalities in stool DNA (22). For the early diagnosis of colorectal cancer, the relevant DNA biomarkers in stool mainly include DNA methylation and mutated DNA. DNA methylation research has received more attention.

Studies have shown that methylated SFRP1 and SFRP2 are significantly associated with CRC (18).A meta-analysis by Yang et al. (23) suggested that the sensitivity and specificity of SFRP2 methylation in the stool of CRC patients were 71% and 94%, respectively. Therefore, methylation of SFRP2 may be significantly associated with CRC. In addition, the methylation of GATA Binding Protein 4(GATA4) is closely related to CRC. Hellebrekers et al. (24) investigated GATA4 methylation in fecal DNA from CRC patients and controls and found it to be 59% sensitive and 88% specific for CRC detection and identified in 42.9% of CRC fecal samples out methylated GATA4. Methylation of the vimentin (VIM) gene encoding the intermediate filament protein vimentin is also commonly used for the non-invasive diagnosis of CRC. One study demonstrated that the sensitivity and specificity of the gene in stool samples for the detection of CRC were as high as 81 percent and 95 percent, respectively (25).In addition to the above target genes, there are some studies confirming that SDC2 (26, 27), SNCA and FBN1 (28), and can be valuable biomarkers for non-invasive detection of CRC.

Compared with DNA methylation, although there are fewer studies on DNA mutations, there is also evidence that it has certain diagnostic value for colorectal cancer. Eighty-five percent of colorectal cancers are caused by chromosomal instability, with mutations accumulating in the E. coli adenomatous polyposis (APC) gene, the p53 tumor suppressor gene, and the K-ras oncogene (29, 30). The other 15% comes from the loss of genes involved in DNA mismatch repair, manifesting as microsatellite instability (31). For example, Traverso et al. (32) detected APC gene mutation in stool samples from 28 non-metastatic CRC patients and 18 patients with adenomas >1 cm in diameter, and found that APC mutation was detected in 26 patients (sensitivity was 57%), while control group was not detected. This suggests that APC gene mutation is closely related to the early events of CRC, and the detection of APC gene may be helpful for CRC screening. Imperiale et al. (30) carried out colorectal cancer screening in 4404 cases of general risk population. Based on this cross-sectional study, a combination of DNA molecular markers containing 21-point mutations was evaluated, and the results showed that the sensitivity of the fecal DNA molecular combination markers for colorectal cancer screening was 52%, while the sensitivity of the guaiac-based fecal occult blood test (gFOBT) was 13% in the same population.

With the development of personalized medicine, the need for precise molecular subtyping of CRC is becoming more and more urgent. In 2015, the International Colorectal Cancer Subtyping Consortium (CRCSC) established a consensus classification of four CRC molecular features (consensus molecular subtype, CMS) - CMS1, CMS2, CMS3 and CMS4) (33). Molecular markers can not only diagnose colorectal cancer, but also assist in the staging and typing of CRC. For example, Gil-Raga et al (34) used BRAFV600E and RAS status and the expression of MLH1 (mutL homolog 1)1and MSH2(mutS homolog 2) to divide 105 patients with stage I-III CRC into five molecular subtypes, and to evaluate clinicopathological features and median survival.

#### 2.2.2 Multi-target stool DNA detection

In recent years, with the deepening of the exploration of tumor molecular diagnostic mechanisms and the continuous progress of stool DNA detection technology, scholars have continued to study gene mutations in the stool. However, the detection of single gene mutation or abnormal methylation in stool cannot accurately reflect DNA variation in intestinal cells. In recent years, a new technology at home and abroad, multi-target stool DNA(MT-sDNA) combined detection technology has emerged and developed rapidly. Compared with the detection of a single gene, this technology can improve the accuracy of diagnosis, and has the advantages of being non-invasive and simple, which offers a new idea and a new method. To obtain better detection efficiency, in 2014, the MT-sDNA kit Cologuard developed by EXAS obtained the first FDA-approved sDNA colorectal cancer early screening product license. Cologuard tests the stool samples provided by the tester and uses the multi-target detection method to analyze the protein and DNA biomarkers related to colorectal cancer and precancerous lesions, mainly including hemoglobin FIT, and DNA site mutation (KRAS gene), DNA methylation.

A study called DeeP-C (35) that included 9989 asymptomatic individuals between the ages of 50 and 84 (who had an average risk of colorectal cancer and were planning to undergo colonoscopy screening) confirmed the efficacy of MT-sDNA in colorectal cancer screening. The fecal samples of the subjects were tested for MT-sDNA and FIT, respectively. The results showed that the detection sensitivity of Cologuard and fecal immunochemical test (FIT) for CRC patients was 92.3% vs 73.8% (P=0.002); the detection sensitivity for serrated sessile polyps ≥1 cm was 42.4% vs 5.1%, respectively. It can be seen that MT-sDNA is better than FIT. However, for patients without colorectal cancer or advanced adenomas, Cologuard was not as accurate as FIT, with specificities of 86.6% vs 94.9%, respectively; for patients with negative colonoscopy results, the specificity of Cologuard and FIT was 89.8% vs 96.4%, respectively. In recent years, Bosch et al. (36)also evaluated MT-sDNA and fecal immunochemical assay (FIT), and the results showed that MT-sDNA assay was more sensitive than FIT alone in detecting advanced precancerous lesions (46% vs 27%, P <0.001). A study from China found that a multi-targeted fecal DNA (MT-sDNA) test was superior to FOBT. This study showed that the sensitivity of FOBT was 19.6% for advanced adenomas and 29.7% for stage I-III CRC. The sensitivity of mt-sDNA detection for adenoma is 50% higher, and the sensitivity for stage I-III CRC is 60% higher (for those who is higher, it is recommended to express clearly). In addition, the sensitivity of mt-sDNA in detecting CRC was 90.0% in the ascending colon, 60.0% in the transverse colon, 75.0% in the descending colon, and 96.8% in the rectum, all better than FOBT (37). Although MT-sDNA has developed rapidly in recent years and is a better screening method for colorectal cancer, it has certain limitations. The test is expensive, and higher costs may limit its use. In addition, there are disadvantages such as low specificity and low detection rate of precancerous lesions.

#### 2.2.3 miRNA

MicroRNAs (miRNAs) are a group of non-coding single-stranded small RNAs (18–22 nucleotides) that inhibit gene expression by binding to the 3′-UTR of target mRNAs. miRNAs are important potential regulators of biological processes and be associated with abnormal expression in diseases such as cancer (38). miRNAs are highly stable and detectable even after long-term storage of fecal samples for several years (39), and thus can be used as potential biomarkers for screening intestinal diseases in feces under disease conditions (40–44). Ahmed et al. (45) found that the expression of seven miRNAs was increased in feces of CRC patients, namely miR-21, miR-106a, miR-96, miR-203, miR-20a, miR-326, and miR-92, while miR-320, miR-126, miR-484-5p, miR-143, miR145, miR-16 and miR-125b expression decreased. Zhu et al. (46) retrospectively analyzed miRNAs in stool samples from 80 CRC patients and 51 controls. The results showed that the expression levels of miR-29a, miR-223, and miR-224 in the feces of the patients in the CRC group were significantly lower than those in the control group (all p<0.001). At the same time, the level of miR-29a (p<0.001) in the stool of patients with rectal cancer was also significantly higher than that of patients with colon cancer. It can be seen that specific miRNAs can be used as biomarkers for CRC screening and early diagnosis (**Table 1**).

**Table 1:** Part of miRNA biomarkers for noninvasive diagnosis of CRC in stool.

micro-RNA	Method	Subjectnumber	Range AUC %	Sensitivity %	Specificity%	References
miR-21	RT-PCR	Cases:40 Control:40	0.829	86.05	81.08	(47)
miR-92a	qRT-PCR	Cases:88 Control:101	–	71.6	73.3	(44)
miR-20a	qRT-PCR	Cases:198 Control:198	0.73	55	82	(81)
miR-29a	qPCR	Cases:40 Control:20	0.777	85	61	(46)
miR-224	qPCR	Cases:40 Control:20	0.745	75	63	(46)
miR-221	miRNA expression array	Cases:198 Control:198	0.73	62.0	74.0	(82)

Most researches on miRNA biomarkers have focused on studying a single miRNA and a single sample type (eg, serum, plasma, or stool). However, in one study (42), after detecting the complementary effect, the combined analysis of miR-223 and miR-92a in the blood and stool of CRC patients obtained higher sensitivity, suggesting that the combined detection of blood and stool miRNA biomarkers could help improve the detection sensitivity of CRC. Wei (43) et al. reported that the AUC of the combination of miRNA-21+miRNA-143 for colorectal cancer was 0.998, the sensitivity was 97.5%, and the specificity was 95.3% so the combination of miRNA-21+miRNA-143 has good diagnostic value for colorectal cancer.

The immunochemical fecal occult blood test (iFOBT) is widely used for colorectal cancer screening; however, its sensitivity is not high. A study exploring a novel colorectal cancer screening method combining iFOBT and FmiRT showed that the sensitivity and specificity of FmiRT using miR-106a were 34.2% and 97.2%, respectively, and the sensitivity and specificity of iFOBT were 60.7% and 98.1%. The overall sensitivity and specificity of the new screening method combining iFOBT and FmiRT were 70.9% and 96.3%, respectively. Therefore, fecal miR-106a can be used to identify colorectal cancer patients from patients with negative iFOBT results. The combination of FmiRT and iFOBT can improve the sensitivity of colorectal cancer detection (47). Some studies have reported the value of circulating miRNAs in detecting patients with metastatic cancer and in CRC, several studies have reported a significant association between circulating miRNAs and metastasis of CRC but the diagnostic utility of fecal miRNAs for CRC metastasis is still unclear. At present, many studies have confirmed that miRNAs can be used as biomarkers for CRC screening and diagnosis. However, its application in clinical evidence is still insufficient, and further large-scale clinical studies are needed to improve the quality of evidence and improve the level of detection technology to make its application more convenient and effective.

#### 2.2.4 Histone modifications

Histone modifications are an important field in epigenetics, and histone modifications include methylation, phosphorylation, acetylation, crotonylation, ubiquitination, glycosylation, ADP ribosylation, etc. Imbalances in histone modifications can lead to tumorigenesis, and loss of methylation and acetylation of histone H3 and H4 residues is a marker of tumor cells (48). Ashktorab et al. (49) studied the expression levels of histone deacetylase 2 (HDAC2) and histone H4K12 and H3K18 acetylation in colorectal cancer (CRC). HDAC2 nuclear expression levels were higher in 81.9%, 62.1%, and 53.1% of CRC, adenoma, and normal tissues, respectively (P = 0.002). The corresponding nuclear total expression levels of H4K12 and H3K18 acetylation were increased in moderately to well-differentiated tumors and decreased in poorly differentiated tumors (P = 0.02). When comparing cancer and non-cancer cases, HDAC2 expression was significantly associated with adenoma progression to carcinoma (P = 0.002), with a discriminative power of 0.74. These results suggest that HDAC2 expression is significantly associated with CRC progression. Another study found that histone H3 lysine 27 acetylation (H3K27Ac) expression was increased in CRC, so it was known that H3K27Ac is related to colon cancer (50). Due to technical limitations associated with quantitative analytes of histone modifications and lack of specificity for different cancers, they have not been used as routine biomarkers for the detection of CRC. Nonetheless, related studies have identified several histone modifications with potential clinical utility as biomarkers for the diagnosis of CRC.

### 2.3 Gut microbiome

**Figure 2:**
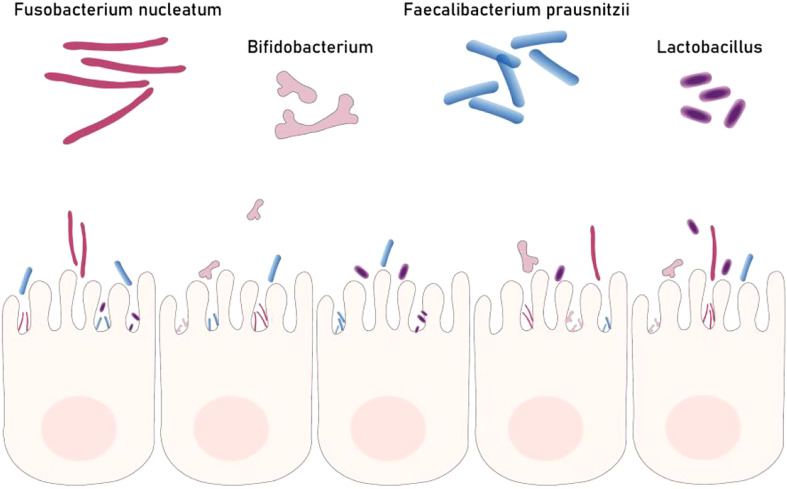
Part of gut bacteria biomarkers for colorectal cancer. Dysbacteriosis is one of the causes of colorectal cancer. Some beneficial bacteria (Fp Bb Lb) can inhibit the occurrence of colorectal cancer but Fn can promote the development of colorectal cancer.

The incidence of colorectal cancer continues to rise and shows a trend of younger people. Compared with genetic factors, it is significantly affected by environmental factors such as improper diet, excessive stress, and excessive use of antibiotics. These environmental factors mainly change the structure of intestinal flora, leading to intestinal microbial dysbiosis that promotes the occurrence and development of colorectal cancer (51). The gut microbiota contributes to cancer mainly through inflammation, immune regulation, dietary component metabolism, and genotoxins. A large number of specific bacteria can be detected in patients with rectal cancer and can be used as biomarkers for disease screening, prediction and treatment response (52) (**Figure 2**).With the development of next-generation gene sequencing and other genomics technologies and metabolomics, the role of gut microbiota in the occurrence and development of colorectal cancer has attracted much attention.

#### 2.3.1 Gut bacteria biomarkers

DAI et al. (53) identified that seven bacteria (Bacteroides fragilis, Fusobacterium nucleatum, Porphyromonas asaccharolytica, Parvimonas micra, Prevotella intermedia, Alistipes finegoldii,Thermanaerovibrio acidaminovorans) are enriched in patients with CRC in a multi-cohort study and these bacteria are thought to be potential markers for diagnosing CRC. A nested case-control study from Sweden investigated 3 bacterial biomarkers in 238 patients. The study found that CLBA+ bacteria and Fusobacterium nucleatum (Fn) were high in feces of CRC patients, and had high specificity (81.5% and 76.9%, respectively) and sensitivity (56.4% and 69.2%, respectively), which can be used for predict cancer (54). Yu et al. (55) identified novel fecal bacterial biomarkers for the diagnosis of colorectal adenomas and cancers. They found through metagenomic analysis that “m3” from Lachnoclostridium, Fusobacterium nucleatum (Fn), and Clostridium hathewayi (Ch) were significantly enriched in adenomas. The sensitivities of m3 and Fn to adenomas were 48.3% and 33.8%, respectively, and the sensitivities to CRC were 62.1% and 77.8%, respectively. In the subgroup tested by the fecal immunochemical test (FIT; n=642), m3 outperformed FIT in detecting adenomas. The combination of m3 with Fn, Ch, Bacteroides, and FIT performed best for diagnosing CRC. In addition, abnormal gut microbiota structure and abundance can be used to judge CRC. Guo et al. (56) measured the relative abundance of Fn, Faecalibacterium prausnitzii (Fp), Bifidobacterium (Bb), and Lactobacillus (Lb) in stool samples from 2 cohorts of 903 individuals and found that the ratio of the abundance of Fn, Bb and Clostridium prazines can be used to predict CRC with a sensitivity of 90%. Multiple meta-analyses have reported that fecal microbes can be used to accurately diagnose CRC. Among them, Thomas et al. (57) verified by cross-cohort that the average AUC of the CRC diagnostic model using the combination of fecal flora as a marker was 0.84, and the pooled analysis of the original metagenome showed that the choline trimethylamine lyase gene was excessive in CRC (P = 0.001), the relationship between microbiome choline metabolism and CRC was determined. Therefore, abnormal gut microbiota structure and abundance may become one of the indicators for CRC screening. Likewise, in a study from Japan, researchers performed a fecal metagenomic test on 616 participants. They found that the relative abundance of Fn increased gradually from intramucosal carcinoma to advanced CRC; two species, Atopobium parvulum and Actinomyces odontolyticus, were found only in multiple polypoid adenomas and/or intramucosal carcinomas (the early stage) increased significantly. This study suggests the potential of using these bacteria as biomarkers for fecal screening (58).

#### 2.3.2 Gut fungi biomarkers

With regard to fungi in the gut, studies have shown that dysbiosis of the fungal flora in the body can be manifested in inflammatory bowel disease (IBD) (59). However, studies on the fungal microbiota in CRC are insufficient.A study from Hong Kong verified the fungal disorders in the feces are associated with CRC. They recruited 184 CRC patients, 197 patients with adenoma, and 204 healthy subjects to represent the intestinal flora in CRC. CRC-related fungal biomarkers and ecological changes were validated in 90 CRC patients, 42 adenoma patients and 66 healthy subjects from Germany and France. The Basidiomycota/Ascomycota ratio was increased in CRC patients compared with healthy individuals. In addition, Malasseziomycetes (fungi) were higher in CRC patients, and Saccharomyces cerevisiae (which has beneficial effects in the gut and has anti-inflammatory properties) and Pneumocystis were depleted. The abundance of 14 fungal biobiomarkers differentiated CRC patients from healthy subjects, suggesting that fungal biobiomarkers may be helpful in diagnosing CRC (60). A new study recruited and sampled 52 patients with newly diagnosed adenoma/CRC and 52 age-matched controls. In this study, researchers found that no bacterial species was significantly associated with CRC, however, interestingly, they observed that the yeast C. albicans was strongly and significantly overrepresented in cases (P = 0.0066, odds ratio 5.444 [95%] CI 1.449–20.462]). Therefore, C. albicans yeast might serve as a potentially valuable screening marker in patients at risk for CRC or in patients with early asymptomatic CRC (61).

#### 2.3.3 Gut viruses biomarkers

Human viruses are prone to mutagenesis and it can modulate their host functionality, so in many cases, it is closely associated with cancer (62).Although many studies have shown a strong link between bacteria and colorectal cancer, the link between viruses and colorectal cancer has not been fully evaluated. Geoffrey et al. (63) found differences in the fecal virome between healthy people and colorectal cancer patients and found that the cancer-associated virome was mainly composed of temperate bacteriophages, suggesting that bacteriophages communities are associated with colorectal cancer and may influence cancer progression by altering bacterial host communities. Similarly, in a study from Hong Kong, researchers used metagenomic analysis finding that the diversity of gut bacteriophages communities was significantly increased in CRC patients compared to controls in fecal samples, which validates that viral markers are associated with CRC. This can be used to detect CRC.

In these years, the term “Oncomicrobiome” (tumor) has begun to be used to refer to this hot research topic. Microbes are closely related to cancer, and numerous studies have confirmed that gut microbes have opened up new avenues for the diagnosis of CRC. However, since there is currently no uniform standard for microbiota detection, standard microbiome biomarker has not been used for CRC detection. Single microbe may not be able to accurately predict CRC, which requires researchers to study multiple microbiome in different ethnic groups of patients for more accurate CRC detection in the future.

#### 2.3.4 Diets or foods factors

Studies have proved that the occurrence and development of colorectal cancer are closely related to diet and foods. One study showed that 38.3% of CRC cases were associated with a poor diet, low in whole grain food intake, low in dairy products, and high in red and processed meat (63). In addition, obesity increases CRC risk by 19%, and being overweight is considered a significant risk factor for CRC (64).

A research team of Harvard Medical School in the United States analyzed the follow-up study data of 134,775 participants in two prospective cohort studies in the United States. During the follow-up period, a total of 3,200 people developed colorectal cancer, which was confirmed by PCR test, of which 1,175 people carrying pks-positive E. coli (pks+ E. coli), they found that a Western-style diet was associated with colorectal cancer containing high levels of pks+ E. coli (people with the highest Western-style diet scores were those with the lowest Western-style diet scores had a higher risk of colorectal cancer 3.45-fold) and not associated with colorectal cancer with low or no pks+ E. coli. The findings suggest that Western-style diets increase colorectal cancer risk through effects on pks+ E. coli (65). Recently, in a large-scale cohort study of young and middle-aged women, the team of Professor Andrew T. Chan found that the intake of processed meat, vegetables and beans is high. Inadequate dietary habits were strongly associated with increased abundance of intestinal sulfur-metabolizing bacteria and were positively associated with the incidence of early-onset colorectal adenomas (66). A Western high-fat diet is one of the risk factors for colorectal cancer. Excess dietary fat can cause a decrease in the diversity of the gut microbiota and a decrease in the abundance of Bacteroidetes and an enrichment of Firmicutes. At the same time, excessive dietary fat can also produce a large amount of secondary bile acids by affecting the metabolism of the flora, increasing the inflammatory response of intestinal cells, DNA damage and the proliferation of intestinal Lgr5(+) cells, thereby promoting the occurrence and development of colorectal cancer (67). Foods or diets can affect the gut microbiota and directly influence bowel contents and stools, thereby promoting the occurrence and development of colorectal cancer. So how foods or diets affect stool and the detection of microbiota in stool to predict colorectal cancer is a promising direction.

### 2.4 Volatile organic compounds

In recent years, the “smell” of diseases has attracted more and more attention, and the identification of diseases by “smell” has become a research hotspot. For example, volatile organic compounds (VOCs) detection has gradually become a new non-invasive early CRC screening method, and some studies have reported some volatile organic compounds as biomarkers for colorectal cancer. A study using stool headspace extraction followed by gas chromatography mass spectrometry to identify volatile organic compounds found that propan-2-ol was significantly increased in cancer samples (P < 0.0001, q = 0.004). The area under the ROC (AUROC) curve is 0.76. When combined with 3-methylbutyric acid, the sensitivity was 87.9% (95% CI 0.87-0.99) and the specificity was 84.6% (95% CI 0.65-1.0), thus volatile organic compounds were important for identifying colorectal adenocarcinoma. It has the superior diagnostic ability (68). Another study reported on the analysis of VOCs in the headspace of stool samples after colonoscopy of subjects to classify them into low-risk (non-cancer) and high-risk (colorectal cancer) groups. By selected ion flow tube mass spectrometry (SIFT-MS) analysis of volatile organic compounds, compared with the low-risk group, it was found that the proportion of hydrogen sulfide, dimethyl sulfide, and dimethyl disulfide in VOCs in the high-risk group was significantly higher (69). As a new type of CRC screening method, VOC is less invasive or non-invasive, with high compliance and high accuracy, but the VOC screening method still needs to be developed, and the detection cost is high. With the continuous development of technology, VOC detection technology is expected to become an important marker to help screen or diagnose colorectal cancer.

## 3 MPE of CRC and biomarker

Molecular Pathology Epidemiology (MPE) is a field of epidemiology based on molecular classification of cancer, which is used to study the influence of molecular characteristics of tumors or other exposure factors on the occurrence and development of tumors (70). MPE is the link between germline genetic and modifiable factors (including environmental, dietary, lifestyle, and pharmacological factors) with pathological features, commonly tumor features (71–73).According to the 2017 World Cancer Research Fund Summary Report (WCRF) and the American Institute for Cancer Research (AICR) based on a systematic review of global studies, concluded that obesity, low physical activity, poor diet (such as more red meat, processed meat, low in fiber, whole grains, and calcium), and alcohol were all associated with an increased risk of CRC (74). Colorectal cancer genome-wide association study reported that genes encoding genes associated with above high risk of colorectal cancer include ATOH1, APOBEC1, BB, BMP5, CDKPN2A, CYP17A1, EIF3H, FKBP5, MED13L, PDLIM5, PTGER4, PTPN1, RTEL1, RPS21, SMARCAD1, SPSB2, TERT or TFEB (75). MPE studies have shown that colorectal cancer patients with a history of smoking are characterized by high microsatellite instability (MSI)-high, CpG island methylator phenotype (CIMP)- high, and BRAF-mutation (76, 77). Bi et al. analyzed the association of KRAS and BRAF gene mutations with pathological features in colorectal cancer patients and reveal the MPE characteristics of colorectal cancer. They pointed out that specific epidemiological characteristics of colorectal cancer patients were associated with KRAS and BRAF mutations. KRAS-mutated tumors are more common in female patients and never smokers. BRAF- or KRAS-mutated tumors are associated with elevated serum levels of colorectal cancer tumor biomarkers (78). Genome-wide association studies (GWAS) reveal interactions between host genetic variation and diet, lifestyle, and other environmental exposures in the development of gastrointestinal cancers (79).A study integrating immunology and MPE into GWAS shows that rs11676348 SNP is associated with colorectal cancer exhibiting Crohn-like lymphoid response or high levels of MSI (80). Combined with the genetic biomarkers mentioned above, future studies can focus on the link between MPE and fecal molecular biomarkers, allowing the detection of more personalized tumor molecular biomarkers in stool.

## 4 Summary and outlook

Early diagnosis of CRC patients is critical, and screening with various non-invasive tests can save lives. The FIT is currently the most commonly used screening tool for CRC screening procedures worldwide. However, FIT is still limited by hemoglobin degradation and an intermittent bleeding pattern, so one-quarter of CRC cases are diagnosed at an advanced stage, resulting in a poor prognosis. Despite the continuous development of endoscopic techniques and the diagnostic level of endoscopists has also continued to improve, the invasiveness and preoperative preparation of colonoscopy; the cost and the lack of popularization of colonoscopy equipment make it difficult for this gold standard to become routine screening for colorectal cancer. As a direct product of gastrointestinal metabolism, stool samples are of great help in the diagnosis of CRC. Fecal biomarkers testing is non-invasive and does not require diet and bowel preparation, which has the advantage of being non-invasive and convenient. Since epigenetics is closely related to tumors, and miRNAs are relatively stable in biological materials. These two types of biomarkers, DNA and miRNAs, are worthy of more in-depth research in the future. In addition, with the current research focus on intestinal flora, fecal intestinal flora also has the potential to be used for colorectal cancer screening and early diagnosis. However, the detection of relevant fecal biomarkers is expensive; the detection technology is immature; the sample size of the relevant test is small, and the results are not universal. In the era of the epidemic, it is inconvenient to go to the hospital for examination, so it is more necessary to develop convenient household non-invasive detection reagents. Fecal biomarkers testing will be a promising non-invasive test for colorectal cancer. In the future, with the gradual maturity and improvement of detection technology, fecal biomarkers combined with fecal occult blood tests are expected to be further developed in the field of screening CRC, becoming one of the main means of colorectal cancer screening, and greatly improving the global level of colorectal cancer prevention and treatment.

## Author contributions

QD contributed to the idea, framework of the review, and write main part of the manuscript. QD is the first authorship. XK contributed to write the abstract and collect some data. XK is the senior authorship. WZ and WL share the corresponding author. They are responsible for reviewing and revising the manuscript. All authors contributed to the article and approved the submitted version.

## Funding

The authors would like to thank the National Natural Science Foundation of China (Grant nos. 82000511), Health Science and Technology Project of Tianjin (TJWJ2021QN006), Scientific Research Project of Tianjin Education Commission (2019KJ197), the Natural Science Foundation of Tianjin (21JCQNJC01120), Tianjin Medical University General Hospital “Excellent Rising Star” Cultivation Project (209060400601) for financially supporting this study.

## Conflict of interest

The authors declare that the research was conducted in the absence of any commercial or financial relationships that could be construed as a potential conflict of interest.

## Publisher’s note

All claims expressed in this article are solely those of the authors and do not necessarily represent those of their affiliated organizations, or those of the publisher, the editors and the reviewers. Any product that may be evaluated in this article, or claim that may be made by its manufacturer, is not guaranteed or endorsed by the publisher.
